# Controlled Dynamic Microfluidic Culture of Murine, Bovine, and Human Embryos Improves Development: Proof-of-Concept Studies

**DOI:** 10.3390/cells13242080

**Published:** 2024-12-17

**Authors:** Jose Roberto Alegretti, Andre M. Da Rocha, Naiara C. Nogueira-de-Souza, Nobuhiro Kato, Bruna C. Barros, Eduardo L. A. Motta, Paulo C. Serafini, Shuichi Takayama, Gary D. Smith

**Affiliations:** 1 Huntington—Medicina Reprodutiva, Av Republica do Libano, 529 Ibirapuera, Sao Paulo 04501-000, SP, Brazil; jalegretti@huntington.com.br (J.R.A.); bcamillo.barros@gmail.com (B.C.B.); emotta@huntington.com.br (E.L.A.M.); paulofiv@terra.com.br (P.C.S.); 2 Department of Gynecology, Federal University of Sao Paulo-UNIFESP, Rua Napoleao de Barros, 715-7°, Sao Paulo 04024-002, SP, Brazil; 3 Department of Obstetrics and Gynecology, University of Michigan, 1500 E. Medical Center Dr., Ann Arbor, MI 48109-0617, USA; moandre@med.umich.edu (A.M.D.R.); ndesouza003@dundee.ac.uk (N.C.N.-d.-S.); nkato@waka.kindai.ac.jp (N.K.); 4 Internal Medicine—Cardiology/Frankel CVC Regeneration Core, University of Michigan, 2800 Plymouth Road, Ann Arbor, MI 48109, USA; 5 Department of Biomedical Engineering, University of Michigan, 2200 Bonisteel Blvd., Ann Arbor, MI 48109-0617, USA; takayama@gatech.edu; 6 Wallace H. Coulter Department of Biomedical Engineering, Georgia Institute of Technology and Emory University School of Medicine, Atlanta, GA 30332, USA; 7 The Parker H. Petit Institute of Bioengineering and Bioscience, Georgia Institute of Technology, 315 Ferst Dr., NW, Atlanta, GA 30332, USA; 8 Department of Urology, University of Michigan, 1500 E. Medical Center Dr., Ann Arbor, MI 48109-0617, USA; 9 Department of Physiology, University of Michigan, 1500 E. Medical Center Dr., Ann Arbor, MI 48109-0617, USA

**Keywords:** prospective randomized controlled trial, microfluidics, murine, bovine, and human embryo culture, dynamic culture, in vitro fertilization (IVF)

## Abstract

Classical preimplantation embryo culture is performed in static fluid environments. Whether a dynamic fluid environment, like the fallopian tube, is beneficial for embryo development remains to be determined across mammalian species. Objectives of these proof-of-concept studies were to determine if controllable dynamic microfluidic culture would enhance preimplantation murine, bovine, and human embryo development compared to static culture. This prospective randomized controlled trial tested static versus controlled dynamic culture of preimplantation mouse (n = 397), bovine (n = 242), and human (n = 512) zygotes to blastocyst stages with outcome measures of embryo cleavage, cellular fragmentation, apoptosis, and blastocyst conversion rates. Dynamic culture of mouse and bovine zygotes with microfluidics significantly improved embryo development. Mouse placental imprinted gene expression was significantly different between embryos derived in vivo, by static culture, and by dynamic culture. Using human sibling zygotes, this dynamic microfluidic culture system increased the number of blastomeres per cleavage-stage embryo, reduced cellular fragmentation or apoptosis, improved blastocyst conversion rates, and enhanced blastocyst developmental stages. In conclusion, species-specific longitudinal studies demonstrated that dynamic microfluidic culture significantly improved embryo development, independent of culture media composition, temperature, and gaseous environment. These cellular indicators represent improved embryo development that can translate into higher pregnancy rates in transgenics, domestic livestock and endangered species and treating human infertility.

## 1. Introduction

**Figure 1:**
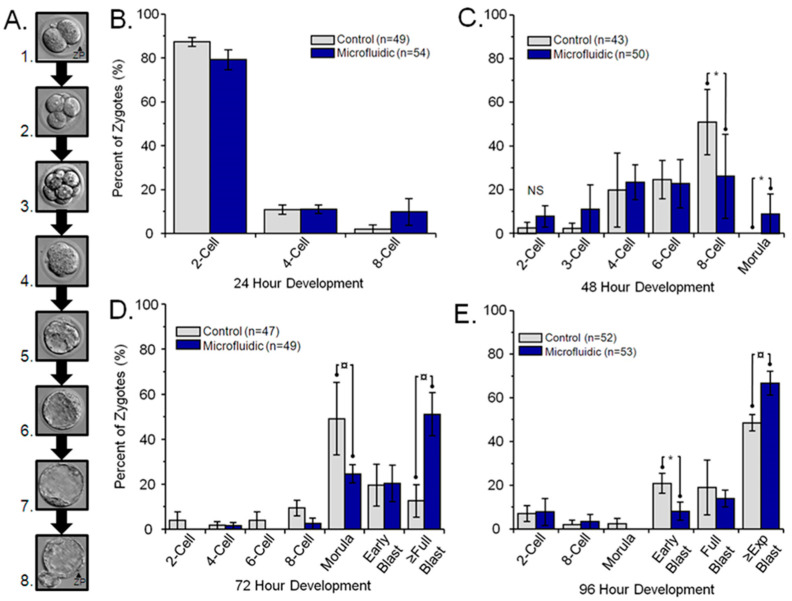
Uninterrupted mouse embryo preimplantation development with and without controlled dynamic microfluidic culture. (**A**) Representative micrographs demonstrating mouse preimplantation embryo development: A.1 (2-cell), A.2 (4-cell), A.3 (8-cell), and A.4 (morula) represent cleavage stages; A.5 (early), A.6 (full), A.7 (expanding [Exp]), and A.8 (hatching) represent blastocyst stages. Mammalian blastocysts implant following complete hatching of the blastocyst from the glycoprotein coat called the zona pellucida (ZP). Mouse zygotes were collected with oviduct flushing and randomly placed into a standard static microdrop culture (Control) or controlled dynamic microfluidic culture (Microfluidic). Zygotes/embryos were cultured in the same media (KSOM 1/2 AA) with the same oil overlay (mineral), temperature (37 °C), gaseous phase (5% CO_2_/Air), humidity (<90%), and incubators in an uninterrupted manner for (**B**) 24 h, (**C**) 48 h, (**D**) 72 h, and (**E**) 96 h. Each graph represents the percent of zygotes reaching the stage of development in set times, with n = number of embryos assessed at each time. Statistical differences in development within a time assessment are indicated as * *p* < 0.05 and ^¤^ *p* < 0.01. Mouse embryos had significantly advanced development in the controlled dynamic microfluidic culture at 48 h compared to controls, which became more significant as embryos progressed through the blastocyst stages at 72 and 96 h of culture.

While the timing of preimplantation embryo development from zygote to blastocyst differs between mammalian species, blastomere cleavages, developmental transitions, and structural/functional characteristics of preimplantation development are similar. Mammalian embryo culture plays a key role in transgenic animal production, genetic enhancement of food-producing livestock, preservation of endangered species, and infertility treatment and fertility preservation in humans. Improved embryonic developmental competence resulting from modifications to the culture environment is quantified by the (1) rate of blastocyst development from the zygote or initial cleavage stage [1]; (2) time-related percentage of embryos developing to a specific blastocyst stage [2] (early, full, expanded, hatching, or hatched blastocyst; Figure 1(A1–A8)); (3) cellular composition of blastocysts (total cell number, ICM, and/or trophectoderm cell count or quality grade) within a set time [3]; and (4) percentage of embryos transferred into the female reproductive tract that implant and establish a live birth [4].

Contemporary mammalian in vitro embryo growth involves culture in media volumes of 4–1000 μL, under static conditions, in plastic petri dishes or test tubes and overlaid with oil [5]. In contrast, in vivo preimplantation embryos develop in the fallopian/oviductal tube in a moist, yet not fully fluid, microenvironment spatially juxtaposed between epithelial cells of oviductal/uterine crypts [6], which are mechanically and chemically dynamic as a result of ciliary movement [7] and segmental muscular contractions [8]. Differences between in vivo and in vitro preimplantation embryo microenvironments can influence embryo development [9,10]. Prototype development and proof-of-concept experiments on microfluidic embryo manipulation and/or culture have been performed with mouse [11,12,13], bovine [14], and human preimplantation embryos [15]. Additionally, numerous theoretical review articles have addressed the potential benefits of microfluidic culture of mammalian embryos [16,17,18]. Our objectives were to perform prospective randomized controlled studies across numerous mammalian species to investigate impacts of precisely controlled dynamic microfluidic culture systems that more closely simulate the in vivo embryo development environment on the development of mouse, bovine, and human embryos.

## 2. Materials and Methods

### 2.1. Microfluidic System

A microfluidic system using polydimethylsiloxane (PDMS) was designed for testing the influence of continual pulsatile fluid flow on embryo development [11]. This microfluidic circuit/cartridge used microchannels as conduits for fluid flow between a medium reservoir and a microfunnel where the embryos resided. A common challenge with PDMS-based microfluidic cartridges is evaporation through PDMS, which was alleviated by using PDMS–Parylene–PDMS membranes [19]. This hybrid membrane minimized evaporation and osmolality shifts yet possessed the thinness and flexibility necessary to interface with deformation-based microfluidic actuation systems. The use of pin actuators that deformed PDMS channels to produce flow eliminated reliance on interconnects, tubing, and external pumps. In this study, on-cartridge pulsatile flow was achieved using computer-controlled, piezoelectric, movable pins on a commercial Braille display (Braillex Tiny, F.H. Papenmeier GmbH & Co. KG, Schwerte, Germany) or a customized variant device [11,20]. An additional challenge in microfluidic systems is bubble formation. To reduce bubble formation, the device assembly process involves plasma treatment, making channels hydrophilic and pre-equilibrating culture media in the incubator before introduction into the device.

### 2.2. Mouse Embryo Culture and Analysis

All mouse procedures were approved by the University of Michigan Animal Care and Use Committee. Eight-week-old B6C3F1 females were given 5 IU equine chorionic gonadotrophin (eCG; Sigma, St. Louis, MO, USA) followed by 5 IU human chorionic gonadotrophin (hCG; Sigma) 48 h later. Females were placed with B6C3F1 males of known fertility overnight and examined the following day for vaginal plugs. Zygotes were isolated from oviducts, with the removal of cumulus cells, and pooled into HEPES-buffered Human Tubal Fluid media with 0.1% Serum Synthetic Substitute (SSS; Irvine Scientific, Santa Ana, CA, USA). Next, 10–15 zygotes were randomly distributed into Potassium Simplex Optimized Medium (KSOM ½ AA with D-Glucose and phenol red, Cat #MR 121-D, Specialty Media, Phillipsburg, NJ, USA) and overlaid with mineral oil (Irvine Scientific) in either (1) 10 µL microdrops (Control) or (2) 10 µL microfunnel culture on PDSM chips with flow-through of fluid at a pin actuation rate of 0.1 Hz that produced an average flow rate of 18 nL/min (Microfluidic). All cultures were performed in the same incubator with a humidified environment of 5% CO_2_ in air at 37 °C. Embryo culture was maintained uninterrupted for 24, 48, 72, or 96 h. At each time point, the device, its chip, and the embryos were removed from the incubator in less than 10 s to minimize temperature and gas fluctuations. Observations at each time point represented a single measurement of embryo development. Embryos were graded in a treatment-blinded manner by two independent observers for cell number at cleavage stages, as morula, or as blastocysts with subdivisions of early, full, expanded, hatching, or hatched.

In a subset of experiments, embryos were cultured for 72 h in PDMS chips without media movement (static culture) or with pulsatile fluid movement (dynamic microfluidics culture). Embryos were graded, and developmentally similar embryos (morula/blastocysts) from each culture condition were transferred into contra-lateral oviducts of pseudo-pregnant recipient mice. As a control, in vivo-derived morula/blastocysts were flushed from donor mice and transferred to recipients. Outcome measures included fetal and placental development and placental imprinted gene expression determined for H19 and IGF2 by quantitative real-time polymerase chain reaction (PCR).

Total RNA extraction was performed with TRIzol^®^ Reagent (Invitrogen, Waltham, MA, USA), and the Rneasy^®^ Mini Kit (QIAGEN, Germantown, MD, USA) was used to purify RNA according to the manufacturer’s instructions. The RNA concentration was determined based on optical spectrometer measurement of absorbance at the wavelength of 260 nm (A_260_). In-uterus-grown blastocysts-derived (in vivo; n = 29), static grown blastocysts-derived (n = 23), and dynamic-grown blastocysts-derived (n = 36) placental total RNA (1 µg each) were reverse transcribed to complementary DNA (cDNA) using GeneAmp^®^ RNA PCR and random hexamers (Applied Biosystems, Waltham, MA, USA) following the standard protocol. Randomly, 20 cDNA from in-uterus-grown blastocysts RNA were pooled and used as a calibrator to compare expression results in quantitative real-time PCR. The individual and pooled cDNA were diluted into 10 ng/μL aliquots and stored at −20 °C. Real-time PCR was performed in a 7300 Real Time PCR System (Applied Biosystems, Foster City, CA, USA). The reaction was performed in 20 μL volumes containing a TaqMan^®^ Universal PCR Master Mix and TaqMan^®^ Gene Expression Assays (Applied Biosystems) following the standard protocol. Five microliters of diluted cDNA (50 ng equivalent) was analyzed for Igf2 (Mm00439564_m1) and H19 (Mm01156721_g1) gene expression. All samples were run in triplicate. The comparative cycle threshold (Ct) was normalized to the glyceraldehydes-3-phosphate dehydrogenase housekeeping gene (Gapdh: Mm99999915_g1) as previously reported [21]. The relative quantification of Igf2 and H19 gene expression was performed using the comparative Ct method [22]. Parametric and nonparametric statistics were performed with ANOVA/unpaired *t*-test or χ-square, respectively. Differences were considered significant at *p* ≤ 0.05.

### 2.3. Bovine Embryo Culture and Analysis

Bovine ovaries were obtained from a local abattoir and transported to the laboratory within two hours of collection at 32–37 °C. Ovaries were rinsed twice with warmed 0.9% saline. Cumulus oocyte complexes (COCs) were aspirated from antral follicles (2–10 mm in diameter) using an 18-gauge needle (Vetpharm, Sioux Center, IA, USA). Only COCs having at least three layers of non-expanded cumulus and an even distribution of cytoplasm were selected. Oocytes were washed three times in HEPES-buffered medium supplemented with 1.0% *v*/*v* PSA (100 units/mL penicillin, 100 μg/mL streptomycin, 0.25 ng/mL amphotericin, Gibco, Grand Island, NY, USA) and once in maturation medium. Selected COCs were matured in tissue culture medium 199 (TCM-199; Gibco), supplemented with 10% fetal calf serum (FCS; Gibco), 0.5 μg/mL bovine follicle-stimulating hormone (FSH), 5.0 μg/mL bovine luteinizing hormone (LH) (Sioux BCHM, Sioux Center, IA, USA), and 10 ng/mL epidermal growth factor (EGF; Sigma). Oocytes were matured in groups of 10 in 50 mL drops of maturation medium covered with mineral oil (Irvine Scientific) at 39 °C in 5% CO_2_ and 100% humidity.

At 22 h post-initiation of in vitro maturation, oocytes were washed three times in warmed HEPES-buffered medium and once in equilibrated fertilization media (IVF-TALP supplemented with 3 mg/mL crystallized bovine serum albumin (BSA), Sigma). Oocytes were transferred in groups of 10 to 50 μL drops of IVF-TALP covered with mineral oil (Irvine Scientific) at 39 °C in 5% CO_2_ and 100% humidity. Frozen sperm from a bull of proven fertility were thawed, and viable sperm were isolated using a 90/45% discontinuous gradient of Isolate (Irvine Scientific) and Sperm-TALP supplemented with fraction V BSA (Sigma). Following isolation, the supernatant was removed, and the sperm pellet was washed and centrifuged in Sperm-TALP supplemented with fraction V BSA. Sperm were counted and used for insemination at a concentration of 1 × 10^6^ sperm/mL of fertilization media. Penicillamine, hypotaurine and epinephrine (PHE) and heparin (5 μg/mL) were added to the fertilization drop to stimulate sperm motility and to facilitate sperm capacitation. Presumptive zygotes were washed three times in HEPES-buffered medium and one time in culture media (KSOM + amino acids supplemented with 3 mg/mL crystallized BSA) and randomly assigned to control or microfluidic cartridges (see microfluidic cartridge set-up below).

Microfluidic cartridges were composed of a thick (~8 mm) PDMS slab with microfluidic channel features, fabricated using soft lithography, attached to a PDMS membrane. The thick PDMS slab with channel features was prepared by casting prepolymer (SYLGARD 184; Dow, Midland, MI, USA) at a 1:10 curing agent-to-base ratio against inside molds composed from an upper mold comprised of silanized PDMS that contained the positive relief features of the channels. The channel relief features were created by casting PDMS against a chemically etched copper printed circuit board prepared by conventional photolithography. The resulting positive relief feature containing the PDMS replica was silanized for use as the bottom PDMS mold. The PDMS prepolymer was cured at 60 °C for 60 min, and holes were made using a dermal punch. The PDMS membrane was prepared using a stepwise procedure of spin coating PDMS onto a 4” silanized silicon wafer to a thickness of 200 µm and curing this layer at 120 °C for 30 min. Embryos were cultured on a 500 µm diameter flat, optically transparent floor. Channels leading into the culture space were 30 µm high and 400 µm wide to prevent embryos from entering the channels. On-cartridge peristaltic pumping was performed using multiple computer-controlled, piezoelectric, moveable pins on a custom Braille display. This pumping motion was set at 0.1 Hz to create a constant exchange of media from the reservoir to the site of cell culture, with an average flow rate of 18 nL/min.

To evaluate the effect of dynamic culture on bovine embryo development, oocytes were matured and inseminated under standard control conditions and presumptive zygotes were randomly transferred into groups of 10 in 50 μL drops of culture media in culture dishes (Control) overlaid with mineral oil or in 50 μL medium overlaid with mineral oil on microfluidic cartridges with dynamic media flow (Microfluidic). Treatment groups were exposed to identical oocyte isolation and insemination strategies, culture media (KSOM ½ AA) with the same oil overlay (mineral), temperature (39 °C), gaseous phase (5% CO_2_/5% O_2_/90% N_2_), humidity (<90%), and incubators. Zygote cleavage was assessed 36 h post-insemination. Blastocyst development was assessed by two independent observers in a treatment-blinded fashion 144 h post-cleavage following uninterrupted embryo culture. Developmental data were analyzed using Chi-square analysis and considered significantly different at *p* ≤ 0.05.

### 2.4. Phase I/Non-Inferiority Clinical Trial of Human Embryo Culture and Analysis

The clinical trial of human embryo culture with conventional methods or dynamic microfluidics was performed at Huntington Medicina Reprodutiva, São Paulo, Brazil in accordance with requirements of the Declaration of Helsinki. This study was approved by the Institutional Review Board of the Federal University of Sao Paulo (UNIFESP), São Paulo, Brazil and by the National Committee for Ethics in Research (CONEP), Process Number 0405/08, Brasília, Brazil.

Couples undergoing in vitro fertilization (IVF) for treatment of infertility were considered for this study. Inclusion criteria were the following: (1) females aged 21–35 years; (2) regular, normal menstrual cycles; (3) normal hormonal profile on day 3 of the menstrual cycle (FSH ≤ 10 IU/mL; LH ≤ 13.5 IU/mL; estradiol (E_2_) ≤ 60 pg/mL); (4) body mass index ≤ 30 kg/m^2^; (5) presence of both ovaries and uterus; (6) maximum of two previous IVF cycle attempts/failures; (7) plans to undergo IVF treatment with autologous oocytes and ejaculated semen; and (8) willingness to participate in the study. Exclusion criteria were the following: (1) females with a history of ovarian hyperstimulation syndrome; (2) patients intolerant to substances used in the treatment; (3) abnormal gynecological bleeding of unknown origin; (4) active substance abuse; (5) clinically significant condition or disease; and (6) if treatment comprised preimplantation genetic testing (PGT). Patients signed informed consent prior to egg retrieval and insemination and were not participating in other trials.

Prior to IVF, female patients underwent controlled ovarian stimulation with gonadotropin-releasing hormone (GnRH) agonist pituitary down-regulation and exogenous supplementation of FSH (Gonal F, Merck Serono, Darmstadt, Germany). Briefly, female patients received daily subcutaneous injections of leuprolide acetate starting at mid- or late luteal phase of the previous menstrual cycle to cause pituitary desensitization. Patients also received recombinant FSH (150–300 IU) daily to promote controlled ovarian follicle growth. The mean (±SE) total dose of FSH used in this patient population for controlled ovarian stimulation was 1989 ± 88 IU, and the mean length of ovarian FSH stimulation was 10 ± 0.2 days. Ovarian follicle and endometrial developments were monitored with ultrasonography every other day, and 250 μg recombinant human chorionic gonadotropin (rhCG; Ovidrel, Merck Serono) was administered when at least two follicles were 18 mm in diameter. Transvaginal ultrasound-assisted oocyte retrieval was performed under mild sedation and analgesia 35–36 h after rhCG injection in an outpatient facility.

Cumulus–oocyte complexes were identified in the ovarian follicle aspirate, isolated, and cultured for four hours after retrieval to allow for final maturation. Oocytes were denuded with brief exposure to hyaluronidase and manual pipetting, followed by washing to remove the hyaluronidase and microscopic assessment for first polar body presence indicating mature metaphase II oocytes. The mean number of isolated mature oocytes was 14 ± 0.6 per patient. Mature oocytes were inseminated with intracytoplasmic sperm injection (ICSI) [23]. At 16–22 h after insemination, eggs were evaluated microscopically for normal fertilization—defined as the presence of two pronuclei (2PN) and two polar bodies.

Couples (N = 45) with ≥8 2PN zygotes consented to participate in this study, in which sibling zygotes on Day 1 (day of zygote assessment) were randomly allocated to culture in static conventional (n = 255) or dynamic microfluidic (n = 257) conditions for 48 or 96 h, resulting in a split-sibling zygote study. Embryos were cultured in the same media, oil overlay, incubators, temperature, and gas phases, and embryo development was assessed on Day 3 or Day 5 (48 or 96 h of culture). While embryo cell number, morphology grading, and blastocyst grading could not be performed blinded to treatment, they were evaluated by two independent individuals, and the results were averaged.

Control (static conventional embryo culture) zygotes/embryos were cultured collectively in a polystyrene organ-culture dish (Falcon 353653, Becton Dickinson, Franklin Lakes, NJ, USA) with 500 μL of medium containing 5% human albumin (G-1 Plus, Vitrolife, Gothenburg, Sweden) under a layer of paraffin oil (OVOIL, Vitrolife) in a humidified incubator at 37 °C, with 7% CO_2_ and air. Embryo culture dishes were prepared 12 h in advance and then placed in the incubator to allow for pre-equilibration, followed by zygote introduction. A maximum of 10 zygotes/embryos were cultured per dish. For the treatment group (dynamic microfluidic embryo culture), the microfluidic cartridge consisted of two independent circuits. Each circuit was composed of two microfunnels linked by two microchannels. Each microfunnel contained 125 µL of medium and was overlaid with paraffin oil. Since microchannels contained ~10 µL of medium, the entire microfluidic circuit contained ~260 µL of medium. To maintain a relative equivalence of zygote- and/or embryo-to-medium ratio, within one circuit, one microfunnel received up to five zygotes for culture, and the other microfunnel contained only medium overlaid with oil, thus serving as a reservoir of fresh media for the dynamic media exchange. Similar to previously described mouse and bovine dynamic/microfluidic culture systems, the bottom of microchannels etched into polystyrene were biocompatible and flexible PDMS/mylar/PDMS sandwich membranes. After loading microfunnels and microchannels with medium (125 µL of medium per microfunnel: G-1 Plus supplemented with 5% of human albumin), cartridges were engaged to an electronic device in which computer-controlled movable pins deformed the PDMS/mylar/PDMS membrane to generate an average medium flow of ~9 nL/min with pulsatile embryo movement and minimal shear stress. Embryo culture dishes were prepared 12 h prior to use, then placed in the incubator to allow for pre-equilibration, followed by zygote introduction. A maximum of five zygotes/embryos were cultured per circuit. Intra-incubator atmospheric conditions were identical to those for static conventional embryo culture.

The following were study participant clinical demographics relevant to assisted reproductive technology (ART) and infertility treatments. Mean body mass index was 22.5 ± 0.4 kg/m^2^, and length of infertility prior to study participation was 2.3 ± 0.2 years. Mean numbers of previous gestations, births, miscarriages, and/or IVF treatment failures (failure to conceive) were 0.3 ± 0.1, 0.0 ± 0.0, 0.3 ± 0.1, and 0.5 ± 0.1, respectively. The representation of infertility etiology within this patient population was 29% male factor, 31% ovulatory dysfunction, 22% tubal factor, 7% endometriosis, 9% multifactorial, and 2% idiopathic.

### 2.5. Morphology Assessment of Human Cleavage Stage Embryos and Blastocysts

Cleavage stage embryos were microscopically evaluated 48 h after placement into control or dynamic culture conditions (Day 3, with Day 0 = insemination and Day 1 = zygote treatment assignment). Blastomeres were counted, and the percentage of cellular fragmentation was determined by two independent observers to collectively provide an average cell number and grade scale for each embryo. Lower grade scale values equate to less cellular fragmentation and superior embryo quality [24]. Briefly, grade 1 represented absence of cellular fragmentation and symmetrical blastomeres; grade 2 indicated up to 10% of total embryo volume consisting of cellular fragmentation and symmetrical blastomeres; grade 3 denoted cellular fragmentation in 11–29% of embryo volume with asymmetric blastomeres; grade 4 signified cellular fragmentation in 30–50% of embryo volume with asymmetric blastomeres; and grade 5 represented an embryo with greater than 50% cellular fragmentation and asymmetric blastomeres. If embryos were selected for an additional 48 h culture, they were transferred to new pre-equilibrated dishes or cartridges that contained the same configurations and volumes, yet with Global medium (Lifeglobal, Guilford, CT, USA) + 10% *v*/*v* protein supplement (LG protein supplement, LifeGlobal) cultured in humidified 37 °C incubators with 5%CO_2_/5%O_2_/90%N_2_. At 96 h of culture (Day 5, with Day 0 = insemination and Day 1 = zygote treatment assignment), embryos were observed for determination of cleavage stage, morula, or blastocyst formation.

Blastocysts were further graded with a slight modification of an established protocol [25] that incorporates a numerical grade of overall blastocyst development and an alphabetical grade for inner cell mass (ICM) and trophectoderm (TE) organization. Briefly, grade 1 represented early blastocysts with blastocoels not fully formed and making up less than 50% of embryo volume; grade 2 equated to expanding blastocysts with blastocoel greater than 50%, less than 100% of embryo volume, and zona pellucida (ZP) not thinning; grade 3 were fully formed blastocysts with the blastocoel filling the entire embryo but no ZP thinning; grade 4 represented expanded blastocysts with the blastocoel filling the entire embryo and the ZP thinning; and grade 5 described blastocysts that were hatching out of, but not fully hatched out of, the ZP. In addition, both the ICM (grade A = many tightly packed cells; B = several loosely grouped cells; C = very few cells) and TE (grade A = many cells in a cohesive epithelium; B = few cells in a loose epithelium; C = very few large cells) were graded, with A representing better than B, and B representing better than C.

Cleavage or blastocyst stage embryos with the best morphology grade were selected for embryo transfer. The selection was independent and blinded to the culture conditions. This study was not designed to evaluate embryo implantation or pregnancy rates. Briefly, the transferred embryos were placed into modified G-MOPS medium (G-MOPS + 50% serum substitute supplement), loaded into a Sydney IVF embryo transfer catheter (Cook IVF, Brisbane, Australia), and transferred into the uterine lumen under transabdominal ultrasound guidance. Serum levels of β-hCG were assessed 12 days after transfer for embryos in the cleavage stage and 10 days after transfer for embryos in the blastocyst stage. Transvaginal ultrasound confirmation of gestation was performed 4 weeks after embryo transfer.

For the statistical analysis, normal distribution and homoscedasticity of data were verified by Kolgomorov-Smirnov test and F test, respectively. Human study sample size was calculated considering means and standard deviations observed in the study with mouse embryos and aimed at a power of 80%. Student’s unpaired *t*-test, Student’s paired *t*-test, and z-test for two proportions were computed as appropriate. Significant differences were attained when *p* ≤ 0.05.

## 3. Results

### 3.1. Mouse Embryo Culture

Mouse embryo development was assessed at 24, 48, 72, and 96 h of uninterrupted culture. Figure 1A represents the embryo developmental stages. While no significant difference was seen at 24 h between culture methods (Figure 1B), a significant (*p* < 0.05) enhancement of mouse embryo development was observed at 48 h of culture (Figure 1C), with more embryos undergoing compaction and morula development (Figure 1(A4)) in the dynamic microfluidic culture group compared to contemporary static control culture. This developmental enhancement became more pronounced (*p* < 0.01) as uninterrupted culture was extended to 72 (Figure 1D) and 96 (Figure 1E) hours. Significantly more embryos reached the full blastocyst stage (Figure 1(A6); *p* < 0.01) after 72 h and the expanded/hatching blastocyst stage (Figure 1(A7,A8); *p* < 0.01) after 96 h of culture in microfluidic compared to static culture.

Mouse embryos derived in vivo (n = 29), in static culture (n = 23), and in dynamic microfluidic culture (n = 36) were transferred into recipients and compared on day 15 of fetal development for (1) fetal morphological age (crown-rump length); (2) fetal weight; (3) placental weight; and (4) placental expression of known imprinted genes H19 and Igf2. Mean fetal morphological age was similar between embryos derived in vivo (14.8 ± 0.2), in static culture (14.7 ± 0.2), and in dynamic microfluidic culture (14.7 ± 0.2). Mean fetal weight was similar between culture groups (static: 260 ± 8.2 mg, dynamic: 266 ± 8.1 mg) but was significantly reduced compared to in vivo controls (294 ± 6.1; *p* < 0.05). Placental weights were not significantly different between groups. Both mean ΔΔCT placental H19 (0.4 ± 0.4) and Igf2 (0.8 ± 0.5) expression were significantly reduced in static culture-derived embryos compared to in vivo-derived embryos (2.2 ± 0.5 and 2.7 ± 0.6, respectively; *p* < 0.05), whereas in dynamic microfluidic culture-derived embryos, only H19 (0.6 ± 0.3; *p* < 0.05) expression was significantly reduced, and Igf2 (1.4 ± 0.5) expression was not significantly different compared to in vivo-derived embryos.

### 3.2. Bovine Embryo Culture

**Figure 2:**
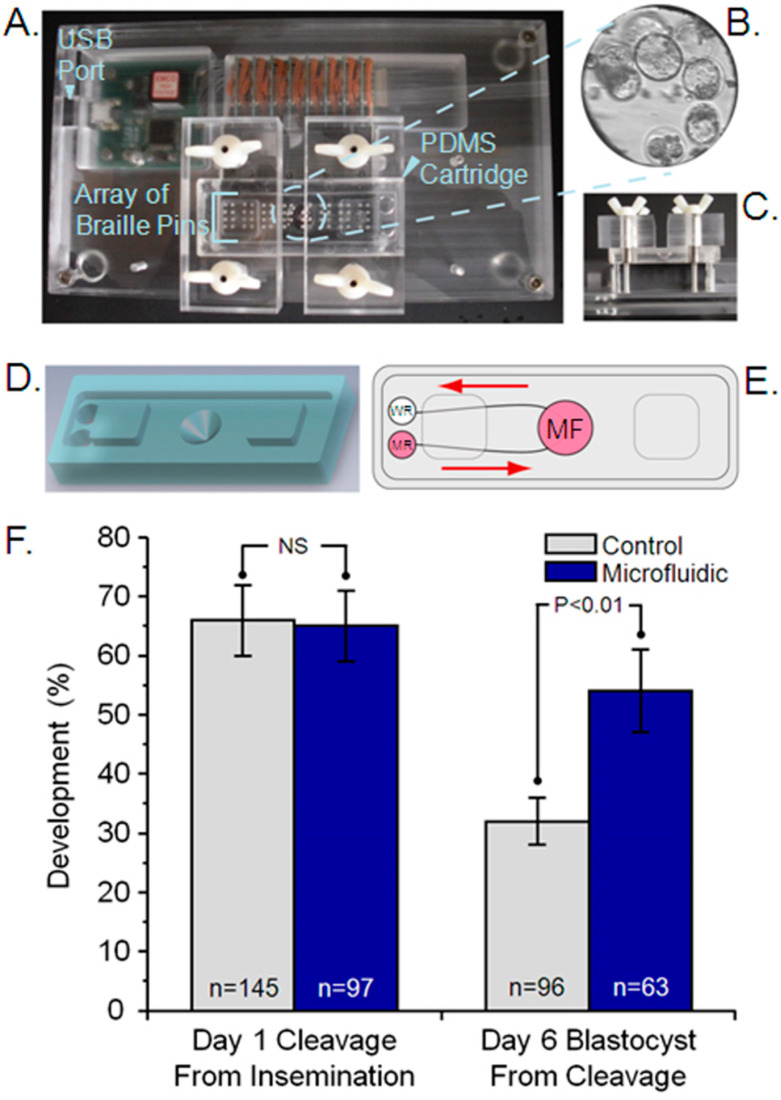
Controlled dynamic microfluidic culture of bovine preimplantation embryos. (**A**) Acrylic resin platforms of 18 cm x 10 cm were constructed to house computer-controlled, piezoelectric, movable pins of a commercial Braille display [20]. Platforms contained a USB port to allow for a cable connection from the inside of the incubator to a laptop computer with software controlling the Braille pin movement outside the incubator. Braille pins were programmed in a five-step pin actuation sequence that generated a pumping motion of 0.1 Hz and continuous pulsatile flow, with average flow rates of 18 nL/min. (**B**–**E**) Microfluidic cartridges were fabricated using soft lithography [27] and composed of a thick (~8 mm) PDMS slab (2.5 cm x 6.5 cm) with microfluidic channel features attached to a PDMS membrane of 200 μm thickness to seal the microfluidic channels [19]. (**B**) Zygotes/embryos were cultured on a 500 µm diameter flat, optically transparent floor at the bottom of a microfunnel-shaped reservoir. (**C**) Cartridges were aligned with microchannel positions above Braille pins and secured with two plastic wing nuts. (**D**,**E**) Schematics demonstrating spatial relations of the microfunnel (MF; embryo placement), microchannels, media reservoir (MR), waste reservoir (WR), and direction of media flow (arrows). Channels leading into microfunnels were 30 µm high and 400 µm wide to prevent embryos from entering the channels. (**F**) Zygote cleavage at 36 h (~1 day) post-insemination and 144 h (~6 days) post-cleavage following uninterrupted culture in conventional microdrops under oil (Control) or controlled dynamic microfluidic culture (Microfluidic). Treatment groups were exposed to identical oocyte isolation and insemination strategies, culture media (KSOM 1/2 AA), oil overlay (mineral), temperature (39 °C), gaseous phase (5% CO_2_/5% O_2_/90% N_2_), humidity (<90%), and incubators in an uninterrupted manner except for a brief (<2 min) evaluation of cleavage. No significant (NS) difference was observed in initial zygote cleavage rates (control n = 145, 66%; microfluidic n = 97, 65%) between culture systems. Following an additional 144 h (~6 days) of uninterrupted culture, the percentage of cleaved embryos (control n = 96; microfluidic n = 63) that developed to the blastocyst stage was significantly enhanced with controlled dynamic microfluidic culture (54%) compared to control (32%; *p* < 0.01). NS = nonsignificant.

In vitro maturation of bovine oocytes, fertilization of bovine mature oocytes on PDMS, and zygote culture for 24 h (Day 1) and initial cleavage (Figure 2F) in dynamic microfluidic culture were equivalent to controls. However, when bovine 2-cell embryos were cultured uninterrupted for 144 h (until Day 6 post-cleavage) in dynamic microfluidic culture, a significant improvement was observed in blastocyst development (54%) compared to conventional microdrop control culture (32%; *p* < 0.01; Figure 2F). The control blastocyst development rate was similar to reported values (39%) of bovine blastocyst development in relation to cleavage within the same time periods [26].

### 3.3. Phase I/Non-Inferiority Clinical Trial of Human Embryo Culture

**Figure 3:**
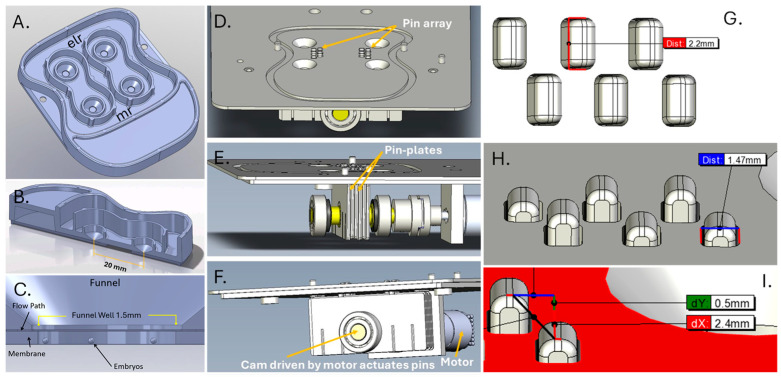
Human embryo culture microfluidic actuator detailed structure and key dimensions. (**A**–**C**) Cut-away of human embryo culture chips with two embryo loading reservoirs (elr)/culture funnels and two media reservoirs (mr), each forming a closed circuit. (**D**–**F**) Cut-away of mechanism driving pins in device (**D**) and an upper angle view showing the pins that drive flow with a part of the cam and plate mechanism inside the device that actuate the pins (**E**). The pins are part of a series of six plates each with two pins that move up and down. (**F**) The pin plates are driven by rotation of a cam that is driven by a motor. The motor-cam-pin mechanism creates a flow of ~20 nL/min at 4 rpm. (**G**–**I**) Pin spacing, general dimensions (2.2 × 1.4 mm), and range of motion (0.5 mm).

The cut-away design of the human embryo culture chips and the mechanics of the pin-flow actuation system are represented in Figure 3. These designs of chip and actuation system were similar to those used in mouse and bovine studies, yet incorporated chip modifications for end-user convenience, and actuator alterations to enhance safe use.

**Figure 4:**
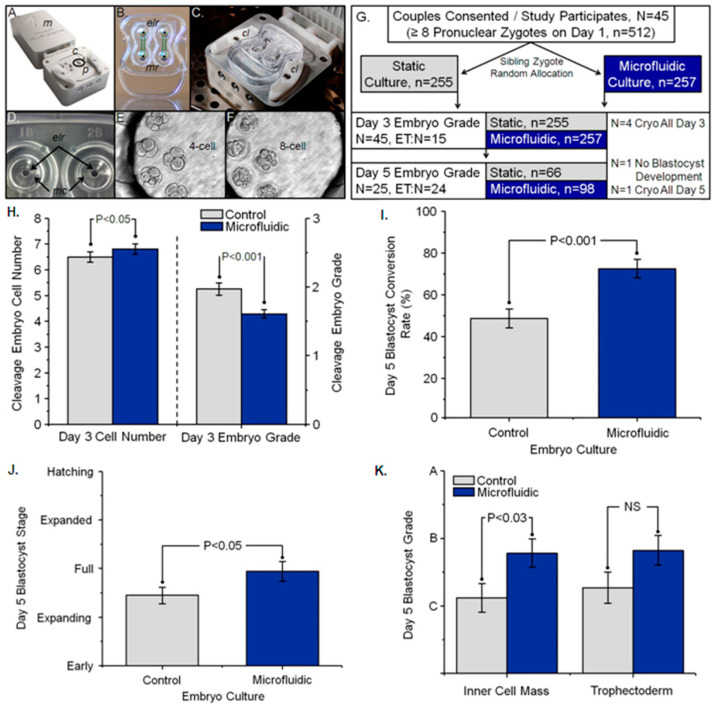
Refinement of platforms and tools, and experimental design of a prospective randomized controlled study to assess the clinical utility of dynamic microfluidic culture of human embryos and resulting embryo development. (**A**) For clinical trial safety, original platforms that housed Braille pin and piezoelectric actuation were replaced with platforms containing a single electric motor (*m*), positioned at a distance from a portion of the platform designed to support the cartridge (*c*). These two portions of the platform were physically separated to facilitate heat dissipation and protection of human embryos. Motors drove a cam shaft that extend from *m* to *c* and contained six oblong lobes/cams, with each cam pushing a row of two pins (one for each circuit) into the flexible membrane on the bottom of the cartridge (**B**). When a pin is pushed up, it acts as a valve to shut a microfluidic circuit. Sequential movement of six pins (*p*) in a predetermined sequence circulates fluid between the embryo-loading reservoir (B-*elr*) and media reservoir (B-*mr*), which are connected by microchannels 30 µm high × 200 µm long—collectively constituting a circuit. A pair of cam levers (*cl*) and tapering posts were designed to hold the cartridge tight on the cartridge platform, enabling pins to generate continual flow between the reservoirs (**C**). A closer view of the embryo-loading reservoirs (D-*elr*) that contained 125 µL of media overlaid with oil (**D**). Entrance of the microchannels (D-*mc*) can be observed at the bottom of the reservoirs. Micrographs (original magnification—200×) of five 4- to 5-cell human embryos on Day 2 (Day 1 = day of randomization and treatment allocation) (**E**) and 8-cell embryos on Day 3 (**F**). Schematic representation of the human embryo trial experimental design. Couples (N = 45; demographics represented in Materials and Methods) with ≥8 normal pronuclear zygotes at time of fertilization assessment (Day 1) consented to participate in this study, resulting in a total of 512 zygotes (**G**). Sibling zygotes from each couple were randomly allocated to either static conventional culture or dynamic microfluidic culture, with a final zygote number per experimental arm of n = 255 and n = 257, respectively. On Day 3, embryos were evaluated for number of cells per embryo and cellular fragmentation grade. Fifteen couples (N = 15) had embryo transfers (ET) on Day 3, terminating their subsequent participation in this study of embryo development to Day 5. In addition, four (N = 4) couples elected to cryopreserve all embryos on Day 3, and one couple (N = 1) had all embryos developmentally arrested in both treatment arms on Day 3 (no blastocyst development). Thus, on Day 5, 25 couples (N = 25) had embryos graded, 24 (N = 24) had blastocyst embryo transfers (ET), and one couple (N = 1) elected to cryopreserve all blastocysts on Day 5. The number of embryos graded as blastocysts on Day 5 for static conventional culture and dynamic microfluidic culture were n = 66 and n = 98, respectively. (**H**) Represents comparisons on Day 3 of the mean (±SE) number of cells per embryo and grade of embryo when zygotes/embryos were cultured for 48 h in control or microfluidic conditions. A significant increase in the number of cells per embryo on Day 3 (approaching 8 cells, considered optimal development) following microfluidic culture compared to control culture (*p* < 0.05) represents improved development. A significantly lower numerical embryo grade on Day 3 (approaching 0, or no cellular fragmentation, considered optimal development) following microfluidic culture compared to control culture (*p* < 0.001) represents improved development. (**I**) Percentage of Day 3 embryos, initiating culture to Day 5, that developed (conversion) into blastocysts. (**J**,**K**) On Day 5, embryos that developed to blastocysts were given a numerical score from 1 to 6 on the basis of their degree of expansion and hatching status (J; see Section 2 for details). In addition, blastocyst inner cell mass and trophectoderm were assigned an alphabetical grade (K; see Section 2 for details), where the grade A is developmentally superior to B, which is developmentally superior to C. NS = nonsignificant.

Zygotes/embryos in dynamic microfluidic culture displayed significantly more cells, less cellular fragmentation, and improved embryo morphology grade than those in static conventional culture after 48 h (Figure 4H). Collectively, this resulted in significantly more Day 3 top-quality embryos (≥8 cells/0% cellular fragmentation) from dynamic culture (38 ± 0.4%) compared to static culture (27 ± 0.4%), *p* < 0.05. The Day 3 to 5 blastocyst conversion rate was significantly improved with dynamic microfluidic culture (77%) compared to static culture (44%), *p* < 0.001 (Figure 4I). Blastocysts resulting from dynamic microfluidic culture were significantly more developmentally advanced (*p* < 0.05; Figure 4J) and had significantly higher quality ICM grades (*p* < 0.03; Figure 4K) compared to blastocysts from conventional static culture.

## 4. Discussion

Initial studies on mouse zygotes/embryos used a controlled dynamic microfluidic culture system previously reported to improve embryo development and implantation [28]. These past studies also demonstrated that mouse embryo culture under dynamic conditions more closely mirrored embryo development in vivo, had implantation rates significantly improved compared to static culture, and closed the implantation rate “gap” between in vitro and in vivo grown embryos. However, in current experiments embryo development was assessed at 24, 48, 72, and 96 h of uninterrupted culture, a practice that is becoming utilized to a greater extent in human ART [29]. Additionally, preimplantation mammalian embryo culture conditions can affect offspring birth weight [30], alter placental imprinted gene expression [31,32], and have been associated with loss-of-imprinted gene disorders [33,34]. Our experiments demonstrate that mouse embryos, when cultured in an uninterrupted manner similar to modern human embryo culture practices, have enhanced development under dynamic microfluidic conditions compared to static culture conditions. Additionally, we found that placental imprinted gene expression can be influenced differentially by static versus dynamic culture conditions during preimplantation embryo development. Whether this change in imprinted gene expression influences preimplantation embryo development is unknown currently.

The bovine zygote/embryo has experimental advantages compared to the murine zygote/embryo in that (1) developmental timing and embryonic genome activation more closely resemble the human embryo; (2) in vitro embryo developmental potential is not as robust and is more sensitive to perturbations in the in vitro environment, which provides a unique opportunity to identify/quantify culture influences; and (3) culture media, conditions, and technical procedures are either specific to the bovine system or more closely replicate human ART practices. We demonstrated that numerous aspects of the bovine embryo in vitro production (IVP) can be performed on microfluidic PDMS cartridges without dynamic culture media (in vitro maturation of bovine oocytes, and initial cleavage of zygotes) in a static media state on a cartridge (Figure 2A–E). Such evaluations are essential for establishing quality control of new materials and systems and confirming the absence of toxicity that can compromise biological materials and experimental testing. Our outcomes demonstrated, for the first time, that important procedures of bovine IVP—including oocyte maturation and insemination—can be performed on a PDMS-devices; and embryo culture can be performed on a PDMS-based microfluidic device with controlled dynamic media flow. One can foresee future studies and benefits of performing multiplexed procedures on a single cartridge/device whereby integration of microfluidic platforms will allow for automation of chemical and mechanical manipulation of gametes and embryos. Microfluidic platforms provide highly controlled and repeatable means to investigate why bovine IVP-produced embryos are sub-optimal compared to their in vivo-derived counterparts [35]. Our studies demonstrate (1) safety of materials and dynamic conditions prior to initiation of human clinical trials; (2) that materials and dynamic culture will support bovine IVP using media, temperature, and gaseous conditions either unique to the bovine culture system or shared with bovine and human embryo culture systems; and (3) that dynamic microfluidic culture improves bovine embryo development compared to conventional static culture.

Our observation of decreased cellular fragmentation in human zygotes/embryos in dynamic microfluidic culture vs. static conventional culture after 48 h, which can represent the degree of apoptosis [36], is of interest because such observations are seldom seen in murine and bovine embryos. This reduced cellular fragmentation along with corresponding significant improvement of cleavage embryo grade may represent reduced sub-lethal stress to the human embryo and an improved culture microenvironment [37]. It has been proposed that embryo fragmentation patterns may represent differential etiologies and effects on subsequent developmental competence [38]. While beyond the scope of this study, future experiments will be enlightening in relation to mechanisms of enhanced development and relation to embryo euploid/aneuploid status. While this study was not designed to compare implantation, pregnancy, or live birth rates, there were numerous instances where human embryos cultured under dynamic microfluidic conditions and transferred on Day 3 or 5 resulted in implantation, pregnancy, and live births. While these observations in this split-sibling zygote human trial are encouraging, it will be important to perform purposefully powered, randomized controlled trials with outcome measures of implantation, pregnancy, and live birth rates.

Use of such culture platforms will also provide general information to improve our knowledge of basic gamete/embryo physiology and developmental biology. Currently, the mechanism(s) responsible for improved embryo development in dynamic compared to static culture has not been fully elucidated. Yet, one can propose that embryo movement in dynamic culture, like in vivo influences in the oviduct, may reduce cell-surface accumulation of metabolic by-products, provide refreshable substrates, and/or provide a combination of factors that does not occur in static culture. Prototypes of microfluidic devices that enable real-time live oocyte/embryo metabolic and secretome analysis, without human manipulation or introduced error, are under development [39,40]. Such live-cell assays will enhance our understanding of normal cellular processes and provide biochemical biomarkers to measure in developmental time and space that are indicative of oocyte/embryo health and viability. Selection of the healthiest embryo for single human embryo transfer based on morphology, genetics, and biomarkers will be facilitated with live-cell bioassays and will reduce incidence of morbidity and mortality associated with multiple gestations in human ART [41]. These culture and selection strategies will enhance the use of elective single embryo transfer (eSET).

## 5. Conclusions

These studies represent longitudinal interdisciplinary experiments encompassing (1) proof-of-concept device design; (2) multi-species testing; (3) device and cartridge development, evolution, and safety evaluation; and (4) the first prospective randomized controlled study comparing controlled dynamic microfluidic and conventional static culture of human embryos. Murine, bovine, and human in vitro embryo development were significantly improved with dynamic microfluidic culture. Cross-species studies demonstrate that in vitro embryo development can be improved with controlled dynamic microfluidic culture in both in vivo and in vitro derived zygotes, with numerous different media, temperatures, and gaseous conditions. Additionally, terminal molecular and epigenetic studies using mouse embryos suggest that, compared to static culture, dynamic culture during preimplantation embryo development can partially normalize placental imprinted gene expression and may influence the health of resulting offspring. Human embryos cultured under dynamic microfluidic conditions resulted in improved development during early cleavage events, reduced cellular fragmentation and apoptosis, an enhanced rate of blastocyst development, and improved ICM grades. These results demonstrate that a programmable controlled dynamic microfluidic culture system can support human embryo preimplantation development; is safe and efficient; and can produce human live births.

## Data Availability

All supporting data for this study and article can be obtained from the corresponding author.
